# Psychological Differences Among Healthcare Workers of a Rehabilitation Institute During the COVID-19 Pandemic: A Two-Step Study

**DOI:** 10.3389/fpsyg.2021.636129

**Published:** 2021-03-31

**Authors:** Anna Panzeri, Silvia Rossi Ferrario, Paola Cerutti

**Affiliations:** ^1^Department of General Psychology, University of Padua, Padua, Italy; ^2^Unit of Psychology-Neuropsychology, Institute of Veruno, Maugeri Scientific Institutes for Research, Hospitalization and Health Care, Veruno, Italy

**Keywords:** assessment, clinical psychology, distress, esteem, healthcare-workers, rehabilitation, COVID-19

## Abstract

**Introduction:** Healthcare workers facing the threatening COVID-19 can experience severe difficulties. Despite the need to evaluate both the psychological distress and positive protective resources, brief and reliable assessment tools are lacking.

**Aim:** Study 1 aimed at developing a new assessment tool to measure psychological distress and esteem in healthcare workers during the COVID-19 pandemic. Study 2 aimed to explore and compare the psychological reactions of healthcare workers of the COVID-19 and the non-COVID-19 wards.

**Methods:** In Study 1, psychologists created 25 items based on their clinical experience. A preliminary qualitative evaluation selected the best 15 items for the new tool (CPI-HP) assessing the COVID-19 psychological impact with 2 scales: psychological distress and esteem. The CPI-HP was administered to 110 healthcare professionals to study its psychometric properties and the internal structure with exploratory graph analysis and confirmatory factor analysis. Study 2 compared two groups of healthcare professionals of the COVID-19 and non-COVID-19 departments.

**Results:** In Study 1, the CPI-HP showed satisfying psychometric properties, and the two-factor structure was confirmed with good fit indices. In Study 2, the two groups of healthcare workers showed comparable levels of psychological distress and resilient coping, but the COVID-19 group displayed significantly higher esteem and appreciation of the experience.

**Discussion:** All operators showed high psychological distress during the emergency, but the COVID-19 group reported higher resources, probably due to stronger group cohesion and greater esteem, perceived meaning, and own work value.

**Conclusion:** Assessing the psychological distress and resources of healthcare professionals with specific tools is important. Psychological interventions should promote their psychological health.

## Introduction

Since December 2019, the COVID-19 disease has rapidly spread, and Italy was one of the first countries in Europe with a vast number of cases. Hospitals and rehabilitation institutes were required to manage and provide care for many acute and post-acute COVID-19 patients while still treating non-COVID-19 patients (e.g., those with cardiovascular and neurological diseases and the elderly).

Health professionals were engaged in the first line to fight the unforeseen, severe, life-threatening, and highly infectious disease and faced several issues. They had to comply with stressful emotional conditions related to both their professional and personal lives. They had to learn new protocols and procedures at work, dealing with an exceeding number of patients and sustaining long shifts with protective clothes with the fear of being infected (Vagni et al., 2020). Also, health professionals' private life was affected as they suffered from psychosocial issues such as isolation and stigmatization; a consistent number of them isolated themselves, moving away from home to not infect their families (Dioscoridi and Carrisi, 2020).

As a whole, these challenging circumstances required prolonged efforts, leading healthcare workers to develop psychological distress symptoms on the cognitive (e.g., worries), emotional (e.g., mood swings), and behavioral (e.g., sleep difficulties and disordered eating) domains (Benfante et al., 2020; Spoorthy, 2020). Despite the COVID-19-related difficulties and the adverse consequences of distress, healthcare professionals were required to cope with the situation, maintaining their individual and professional functioning (Di Tella et al., 2020).

Some key protective factors may have helped healthcare professionals to cope with the situation in a resilient way.

According to the anxiety buffer hypothesis (ABH) (Greenberg et al., 1992; Rossi et al., 2020a), (self-)esteem can represent a resource and a protective factor buffering the effects of stress. Indeed, as stated by the terror management theory (Pyszczynski et al., 2004), esteem also relies on one's social role and is reinforced by other society components. In the COVID-19 pandemic, health operators felt they had a crucial role in the emergency. By doing a meaningful and important job at a societal level, they felt more valuable and important, and their esteem was strengthened by the support and recognition provided by family, colleagues, and society (Greenberg et al., 1986; Hennekam et al., 2020). Moreover, according to the social identity theory (SIT; Tajfel and Turner, 1986), people can derive a sense of self-worth and social belongingness from their memberships in groups. In other words, in the COVID-19 emergency, the perception of being part of an (in-)group of peers and colleagues (i.e., the work team) sharing common features, aims, and feelings and supporting each other could have represented another factor sustaining esteem against distress.

Besides, a resilient coping strategy may have helped healthcare professionals to preserve good functioning despite the distress. *Coping* is the process of facing, tolerating, and reducing stress related to the demands of an adverse circumstance—as the pandemic—triggering negative emotions (Kocalevent et al., 2017). Individuals using resilient coping strategies can control their responses to stress and react to difficulties positively (Sinclair and Wallston, 2004). While some individuals are more prone to perceive the negative aspects of a situation (Giuntoli et al., 2019), others can still appreciate positive aspects even in the worst circumstances and show the so-called post-traumatic growth (Chen et al., 2020).

Given this background, in the critical and challenging circumstances represented by the outbreak of infectious disease, it is important to assess and monitor the psychological health of healthcare professionals to support them in coping with stress.

However, to date, there are no specific tools specifically developed to measure the psychological impact of COVID-19 on healthcare workers. Most of the existing studies used preexistent tools, mostly related to the negative impact of events, anxiety, and depression (Benfante et al., 2020; Pappa et al., 2020). Thus, a brief tool specifically developed to measure the healthcare professionals' psychological distress and esteem when facing the COVID-19 emergency was lacking.

Moreover, given the frequently asymptomatic and undetected COVID-19 infections, the healthcare workers of both COVID-19 and non-COVID-19 wards were exposed to stressful conditions, potentially triggering similar distress levels. Nonetheless, the similarities and differences in the psychological characteristics of health professionals working in COVID-19 and non-COVID-19 wards were not yet explored. Although similar distress levels are expected, the contribution of protective factors may differ among these groups, namely, the group cohesion and the perceived esteem.

### The Present Research

The present two-step research aimed to measure and explore the psychological experience of healthcare professionals in a rehabilitation institute in the north of Italy, where a dedicated ward for patients with COVID-19 was opened in the middle of March 2020.

In Study 1, a new assessment tool was developed to evaluate the psychological impact of COVID-19 for healthcare workers, and its psychometric properties were analyzed.

In Study 2, the questionnaire created in Study 1 was used to assess, explore, and compare the psychological distress and adaptation of health workers and employees working in COVID-19 and non-COVID-19 wards of the institute.

## Study 1

Study 1 aimed to develop an *ad hoc* self-report questionnaire assessing the most relevant psychological areas across healthcare workers' experience during the COVID-19 pandemic—(A) psychological distress and (B) esteem—and to evaluate its psychometrical properties.

### Methods

#### Participants and Procedure

Participants of the study were recruited in a rehabilitation center in northern Italy. All the healthcare workers received an institutional e-mail presenting the study and inviting them to participate in it. Then, the coordinators of each section renewed the invitation to the study. Interested workers were invited to contact the psychologists.

Inclusion criteria were (I) being a native Italian speaker and (II) working in the rehabilitation center during the COVID-19 emergency; and the exclusion criterium was (III) not being able to complete the questionnaire. The final sample of this study was composed of 110 healthcare workers [31 (28.2%) males and 79 (71.8%) females] aged from 23 to 66; mean age = 44.13 (*SD* 11.17)]. The sample characteristics are reported in Table 1.

**Table 1:** Study 1 and Study 2: descriptive statistics of the samples.

	**Study 1**	**Study 2**		
	**Total (*N* = 110)**	**Total (*N* = 68)**	**COVID-19 ward (*n* = 34)**	**Non-COVID-19 ward (*n* = 34)**
Age, mean (SD)	45.70 (10.80)	40.409 (11.250)	39.719 (11.191)	41.059 (11.433)
**Sex**, ***n*** **(%)**				
Males	20 (22.22%)	28 (41.18%)	15 (44.12%)	13 (38.24%)
Females	70 (77.78%)	40 (58.82%)	19 (55.88%)	21 (61.76%)
**Marital status**, ***n (%)***				
Single	23 (28.7%)	22 (32.35%)	6 (17.65%)	16 (47.06%)
Married	44 (48.9%)	37 (54.41%)	21 (61.76%)	16 (47.06%)
Separated/divorced	12 (13.3%)	6 (8.82%)	5 (14.71%)	1 (2.94%)
Widow	1 (1.1%)	–	–	–
**Education**, ***n (%)***				
Middle school	8 (10%)	7 (10.29%)	6 (17.65%)	1 (2.94%)
High school	21 (26.3%)	13 (19.12%)	8 (23.53%)	5 (14.71%)
Degree	47 (58.8%)	44 (64.71%)	19 (55.88%)	25 (73.53%)
Master/specialization	4 (5%)	4 (5.88%)	1 (2.94%)	3 (8.82%)
**Professional role**, ***n (%)***				
Healthcare assistant	9 (10.8%)	8 (11.76%)	6 (17.65%)	2 (5.88%)
Professional nurse	36 (43.4%)	25 (36.76%)	12 (35.29%)	13 (38.24%)
Rehabilitation technician	16 (19.3%)	10 (14.71%)	4 (11.76%)	6 (17.65%)
Physician	9 (10.8%)	10 (14.71%)	5 (14.71%)	5 (14.71%)
Administrative	6 (7.2%)	5 (7.35%)	–	5 (14.71%)
Maintainer	1 (1.2%)	5 (7.35%)	5 (14.71%)	–
Other	6 (7.2%)	5 (7.35%)	2 (5.88%)	3 (8.82%)
**Psychological measures, mean (SD)**				
Psychological distress	21.178 (6.989)	19.147 (7.184)	17.059 (6.415)	21.235 (7.394)
Esteem	20.600 (6.900)	21.118 (7.074)	23.618 (6.527)	18.618 (6.791)
Coping	9.9444 (2.628)	10.176 (2.823)	10.147 (3.036)	10.206 (2.637)
Experience	57.614 (24.436)	62.879 (24.164)	73.750 (21.137)	52.647 (22.537)

Healthcare workers completed informed consent, a demographic measures form, and the items of the new questionnaire. This research was conducted according to the Helsinki guidelines and was approved by the Scientific Direction of the Institute. All participants were informed about the study aims and voluntarily agreed to participate.

#### Sample Size Calculation

Considering statistical analyses used in this study (see the designated section), scientific literature guidelines suggest that exploratory analysis could correctly estimate model parameters with a minimum sample of 100 observations (Golino and Epskamp, 2017). Moreover, also for simple confirmatory models, 100 individuals were considered adequate (Marsh et al., 1988; Kelloway, 2015).

#### Measures

##### Development of the COVID-19 Psychological Impact-Healthcare Professionals

The item pool for the CPI-HP was developed using a three-step double-blind study procedure—already employed in other studies (Simpson et al., 2018; Milavic et al., 2019; Pietrabissa et al., 2020a,b).

*First*, two psychologists–psychotherapists (SRF and PC) who supported healthcare workers during the first phases of the pandemic independently created a pool of items to assess the 3 scales: (A) *psychological distress* and (B) *esteem of healthcare workers—*focusing attention on constructs coverage. The *psychological distress* dimension concerned the fear and anxiety of being infected, mood swings, irritability, and helplessness. Psychological distress included not only emotional facets but also beliefs and behaviors. The *esteem of healthcare workers* dimension referred to other- and self-perceived personal values, motivation to work, and belongingness to one's workgroup.

*Second*, the two lists of items were compared and screened: item phrasing was adjusted for the target population, and redundant items were removed. Thus, a preliminary item list (25 items) was approved by SRF and PC.

*Third*, a third psychologist (AP) administered the list of items to a sample of 10 healthcare workers (*judges*)—who sorted (in order of relevance) the most representative items for each dimension—giving attention to relevance and comprehensibility. Conclusions from the judges were matched and discussed. An agreement higher than 90% between judges was considered adequate to retain the item. If an agreement was reached for more than one item per dimension, judges were asked to select the most significant one. Finally, a list of 15 items (eight for *psychological distress* and seven for *esteem*) was provided.

Items were scaled on a 5-point Likert-type scale ranging from 0 (*never*) to 4 (*always*). The total score of each dimension (*psychological distress* or *esteem*) was computed by summing the items of each factor. The higher the score, the higher the value in that scale—thus the higher the psychological distress and/or esteem. No overall total score (psychological distress *plus* esteem) should be calculated. In the Appendix, Table A1 shows the 15 items of the questionnaire.

#### Statistical Analysis

The R software (R Core Team, 2017) was used with the following packages: bootnet (Epskamp et al., 2018), EGAnet (Golino and Christensen, 2020), mgm (Haslbeck and Waldorp, 2020), lavaan (Rosseel, 2012), and psych (Revelle, 2018).

First of all, the level of item informativeness was checked (Mullarkey et al., 2018, 2019; Marchetti, 2019). Each item was compared to the mean level of informativeness of the CPI-HP (0.078) plus/minus 2.5*SD*s (0.194). Poorly informative items were excluded from subsequent analyses.

Second, an exploratory graph analysis (EGA) (Golino and Epskamp, 2017; Giuntoli and Vidotto, 2020; Golino and Christensen, 2020) was performed to assess item clustering by using the walktrap algorithm for weighted networks (Pons and Latapy, 2006)—in which nodes may cluster together forming tidy connected sub-networks. Consequently, the thicker an edge, the strongest the relationship between the items of a specific cluster (dimension/factor) (Mair, 2018; Christensen and Golino, 2020). Moreover, it has been demonstrated that EGA has an almost perfect accuracy to correctly extract the correct number of dimensions of a questionnaire—also with a sample size of 100 individuals (Golino and Epskamp, 2017).

Third, to confirm the results of the EGA (Chandrasekaran et al., 2012; Costantini et al., 2015; Epskamp et al., 2017), a confirmatory factor analysis (CFA) was performed (Christensen and Golino, 2020). Considering the CPI-HP response scale, the diagonally weighted least square (DWLS) estimator was used to perform each CFA (Brown, 2015; Lionetti et al., 2016; Manzoni et al., 2021). The model fit of the factorial structure of the CPI-HP was assessed through the (A) Satorra-Bentler χ^2^ (S-Bχ^2^); (B) root-mean-square error of approximation (RMSEA); (C) comparative fit index (CFI); and (D) the standard root mean square residual (SRMR) (Muthén, Muthén, 1998–2017; van de Schoot et al., 2012; Brown, 2015; Kline, 2016). The following cutoffs for “acceptable” model fit were applied: the S-Bχ^2^ should be non-statistically significant (*p* > 0.05); the RMSEA should be lower than 0.080; the CFI should be higher than 0.90; and the SRMR should be lower than 0.080 (Hu and Bentler, 1999; Hoyle, 2012; van de Schoot et al., 2012; Brown, 2015). The internal consistency of each scale was assessed with Cronbach's α.

The adjusted item–total correlation was also calculated. Also, given that the CPI-HP is a new instrument, the items' ability to discriminate subjects with low or high scores was tested (Milavic et al., 2019; Consoli et al., 2020; Pietrabissa et al., 2020a); thus, the item discriminant power (IDP) was computed (Ebel, 1965; Chiorri, 2011). According to the literature about typical performance test items such as Likert scales, the maximum total score and the quartile rank were calculated for each participant. Then, the item discriminating power was calculated by using independent-sample *t*-tests and Cohen's Cohen (1988) *d*, the dependent variable was the total score of each scale, and the grouping variables were the lowest and the highest quartiles (Ebel, 1965; Chiorri, 2011; Milavic et al., 2019; Consoli et al., 2020; Pietrabissa et al., 2020a).

### Results

#### Preliminary Analysis

As reported in Table 2, all the items were almost normally distributed, and none of them was poorly informative. Thus, all 15 items used to compose the CPI-HP could be retained into the principal statistical analyses for assessing the dimensionality of the questionnaire.

**Table 2:** Study 1: psychometric properties of items.

	**Descriptive statistics**				**ITC**	**IDP**		**EGA**	**CFA**	
	***Mean***	***SD***	**Skewness**	**Kurtosis**	***r***	***t***	***d***	***dim***.	**λ**	***R*^**2**^**
Distress	15.93	6.443	−0.193	−0.671						
Item #1	2.12	1.247	−0.257	−0.959	0.423	−8.490	2.413	1	0.505	0.255
Item #2	1.61	1.134	−0.064	−1.126	0.397	−6.141	1.805	1	0.444	0.197
Item #3	2.21	1.084	−0.384	−0.652	0.736	−12.546	3.590	1	0.809	0.655
Item #4	1.94	1.294	−0.164	−1.132	0.595	−8.337	2.428	1	0.702	0.493
Item #5	2.03	1.121	−0.174	−0.681	0.786	−10.316	2.955	1	0.926	0.858
Item #6	2.73	1.031	−0.657	0.107	0.450	−5.859	1.668	1	0.507	0.257
Item #7	1.83	1.180	−0.204	−1.128	0.667	−10.862	3.109	1	0.829	0.688
Item #8	1.47	1.232	0.439	−0.657	0.588	−7.880	2.319	1	0.729	0.532
Esteem	16.30	6.222	−0.296	−0.197						
Item #1	2.68	1.092	−0.586	−0.180	0.509	−6.679	1.889	2	0.576	0.332
Item #2	2.25	1.137	−0.289	−0.606	0.689	−10.120	2.859	2	0.765	0.585
Item #3	2.52	1.115	−0.330	−0.573	0.645	−8.983	2.536	2	0.761	0.579
Item #4	2.30	1.138	−0.082	−0.762	0.647	−12.122	3.443	2	0.746	0.557
Item #5	2.38	1.226	−0.405	−0.770	0.755	−13.496	3.774	2	0.842	0.709
Item #6	1.77	1.290	0.226	−1.033	0.699	−18.203	5.180	2	0.812	0.660
Item #7	2.39	1.150	−0.299	−0.523	0.711	−10.363	2.906	2	0.788	0.620

*ITC, item total correlation; IDP, item discriminant power; d, Cohen's d; EGA, exploratory graph analysis; dim., dimension resulting from EGA; CFA, confirmatory factor analysis; λ, item factor loading; R^2^, explained variance*.

#### EGA

**Figure 1:**
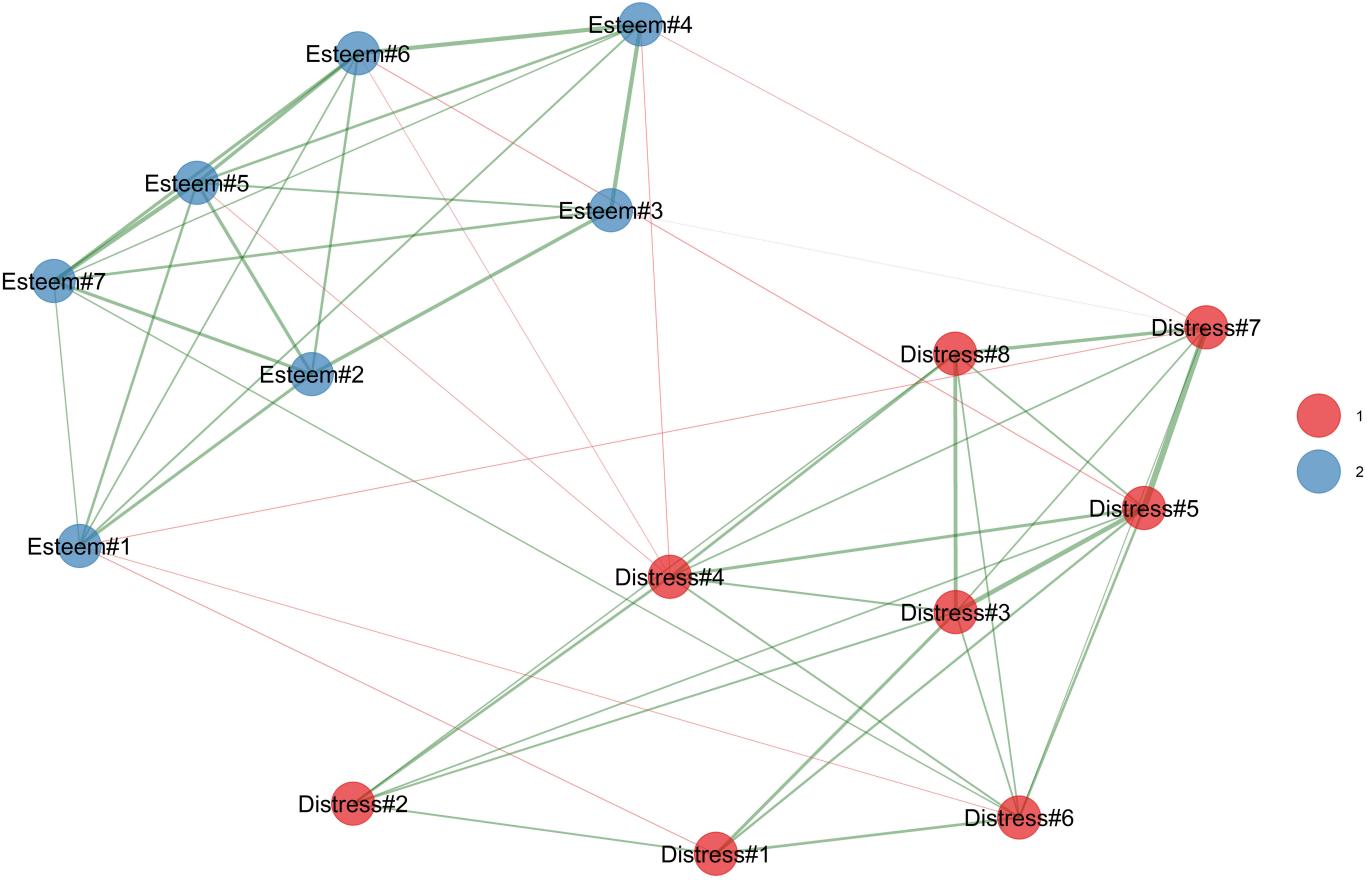
Study 1. Exploratory graph analysis of the CPI-HP.

As reported in Figure 1, the EGA strongly confirmed the hypothesized two-factor solutions. Indeed, two well-separated sub-networks were identified in the CPI-HP network structure. In particular, on the one hand, the *psychological distress* was in red, and it was composed of all of the supposed 8 items. On the other hand, the (B) *esteem of healthcare workers* was in blue, and it was composed of all of the supposed seven items. These results suggest the two-factor-related first-order factor dimensionality of the CPI-HP scale.

#### Structural Validity

As showed in Table 2, the CFA clearly confirms the EGA results. Indeed, a good solution for the CPI-HP was found: S-Bχ^2^(89) = 72.772; *p* = 0.894 *ns*; RMSEA = 0.000 [90% CI: 0.000–0.025; *p*(RMSEA ≤ 0.05) = 1 *ns*]; CFI = 1.000; SRMR = 0.065. In addition, model modification indices showed that the model could not be improved. The two factors showed a small negative correlation: *r* = −0.321.

For the *psychological distress* scale, item factor loading ranged from 0.444 (item #2) to 0.926 (item #5): (*mean* = 0.681, *SD* = 0.177), with the items' *R*^2^ ranging from 0.197 to 0.858 (*mean* = 0.492, *SD* = 0.239). For the *esteem of healthcare workers* scale, item factor loading ranged from 0.576 (item #1) to 0.842 (item #5) (*mean* = 0.756, *SD* = 0.086), with the items' *R*^2^ ranging from 0.332 to 0.709 (*mean* = 0.577, *SD* = 0.120).

#### Psychometric Properties

Regarding internal consistency, Cronbach's α coefficients for the questionnaire scales were good: for the *psychological distress* scale, α = 0.842, and for the *esteem of healthcare workers* scale, α = 0.880.

The IDP analysis showed that 15 items of the CPI-HP discriminated well between subjects with low and high forgiveness of self, other, and situation in both first- and second-order dimensions (Table 1). Considering the *psychological distress* scale: the lower discriminative item was item #6 (*t*_i_ = −5.859, *p* < 0.001, *d* = 1.668), and in the opposite, the higher discriminative item was item #3 (*t*_i_ = −12.546, *p* < 0.001, *d* = 3.590). Considering the *esteem of healthcare workers* scale, the lower discriminative item was item #1 (*t*_i_ = −6.679, *p* < 0.001, *d* = 1.889), and in the opposite, the higher discriminative item was item #6 (*t*_i_ = −18.203, *p* < 0.001, *d* = 5180).

Finally, the adjusted item–total correlation showed statistically significant negative associations between each item and their respective factors (Table 1).

## Study 2

### Method

Study 2 aimed to assess and compare the psychological experience of the health professionals who worked in COVID-19 and non-COVID-19 wards of the institute.

Inclusion criteria were (I) being a native Italian speaker and (II) working in the rehabilitation center during the COVID-19 pandemic; the exclusion criteria was (III) not being able to complete the questionnaire.

Thus, a sample of 68 employees and health workers of the institute was considered. Half of them worked in the COVID-19 ward, while the other half did not. The two groups were strictly matched for age, sex, and professional role. It is worth noting that the COVID-19 group differed in terms of support of the professional activity (e.g., more strict hygiene protocols and more protections) and enhanced by a Whatsapp support group among colleagues of the work-team—these aspects may have a positive effect on the psychological experience.

The final sample of this study was composed of 68 healthcare workers [28 (41.18%) males and 40 (58.82%) females] aged from 23 to 62; mean age equal to 40.41 (*SD* = 11.25)]. The sample characteristics are reported in Table 1.

Participants gave informed consent and completed a questionnaire including demographics and psychological measures. Also, this research was conducted in agreement with the Helsinki guidelines, it was approved by the Scientific Direction of the Institute, and all participants voluntarily agreed to participate and provided written informed consent.

### Measures

#### CPI-HP

The CPI-HP questionnaire—created in Study 1—was administered to evaluate the *psychological distress* with 8 items and *esteem* with seven items (total 15 items). The response format was a 5-point Likert-type scale from 0 (*never*) to 4 (*always*). Higher scores on each scale indicated higher levels of the measured variable. The α in this study was 0.830 for distress and 0.874 for esteem.

#### Brief Resilient Coping Scale

The BRCS (Sinclair and Wallston, 2004) is a four-item self-report tool to measure resilient coping, defined as the tendency to cope with stress in a highly adaptive and positive way despite the difficulties. The response format is a 5-point Likert-type response form (1 = “the statement does not describe me at all” and 5 = “it describes you very well”). Scores range from 0 to 16, with higher values indicating more resilient coping. The BCRS showed good internal consistency in this study, where the α was 0.72.

A visual analog scale (VAS) called *positivity of experience* asked participants to rate the degree of appreciation of their work experience during the COVID-19 pandemic from extremely negative (0) to extremely positive (100).

### Statistical Analyses

The R software was used (R Core Team, 2017) with the following packages: *esvis* (Anderson, 2020), *ggplot2* (Wickham, 2016), *overlapping* (Pastore and Calcagnì, 2019), and *psych* (Revelle, 2018).

Similarities and differences among these groups were studied using independent-sample *t*-tests. Besides, Hedge's (1981) *g* was used as the effect size according to the guidelines' thresholds. Hedges' *g* is interpreted similarly as Cohen's *d*; the following rule of thumb can be used to interpret the results: 0–0.2 = *small* effect (not visible to the naked eye); 0.5 = *medium* effect; and 0.8–1 = *large* effect (visible to the naked eye). Moreover, Hedge's *g* was supported by the overlapping index (η); that is, it was used to quantify the magnitude of differences between the Kernel density distributions of the groups (Huberty and Lowman, 2000; Wen and Fan, 2015; Pastore and Calcagnì, 2019; Rossi et al., 2020b). The η ranges from 0 (perfect separation) to 1 (perfect overlap); thus, it should be interpreted as other normalized effect sizes (i.e., explained variance and percentage) (Pastore and Calcagnì, 2019).

### Results

**Figure 2:**
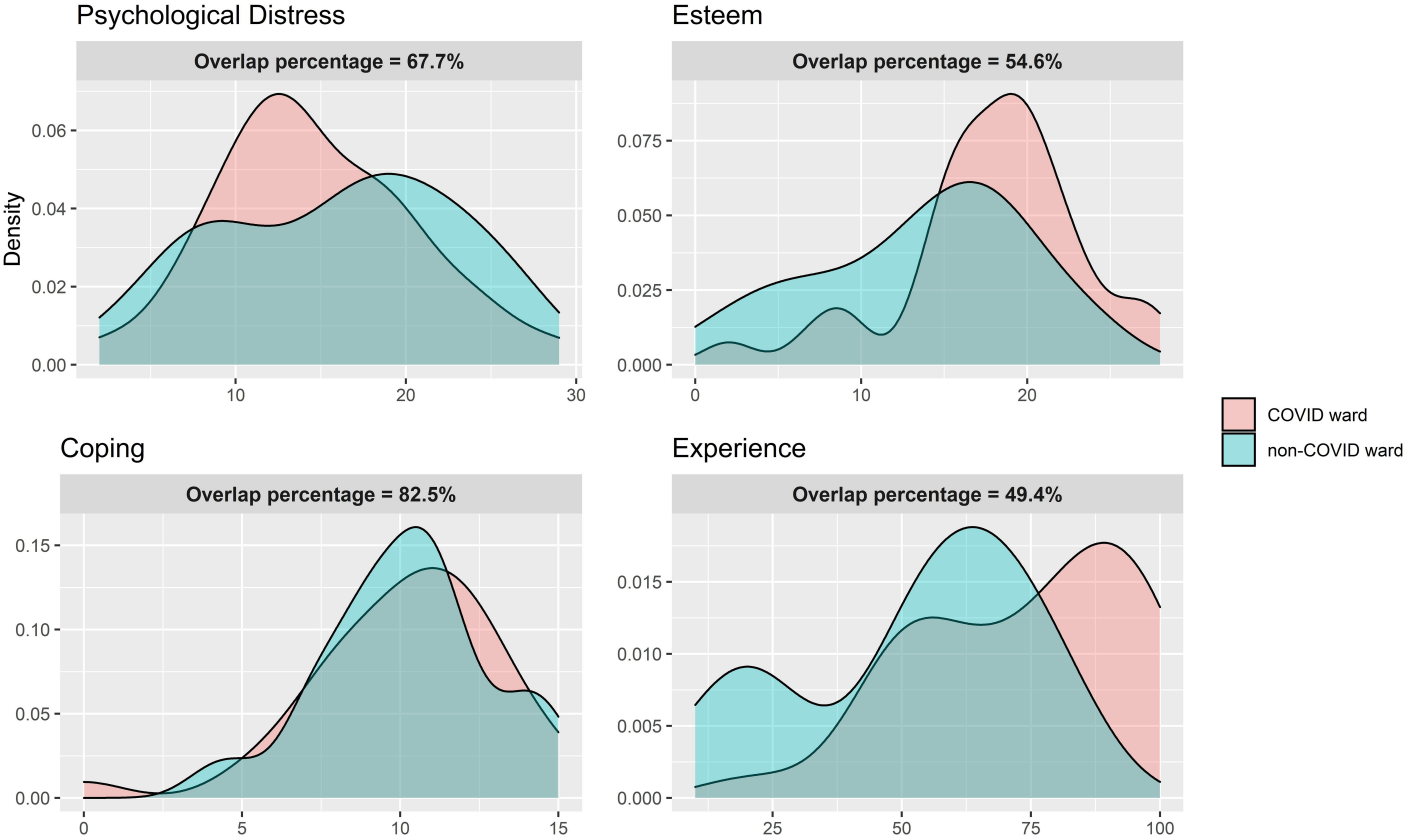
Study 2. Distributions overlapping between HC from the COVID-19 ward and HC from the non-COVID-19 ward.

The psychological measures of the two groups are reported in Table 2; Figure 2 shows the overlapping graphs.

#### Psychological Distress

According to the Welch two-sample *t*-test, both groups reported comparable levels of psychological distress (COVID-19 group: mean = 14.68, *SD* = 7.03; non-COVID-19 group: mean = 15.911, *SD* = 15.91) with a non-statistically significant difference [*t*_(63.96)_ = 0.787, *g* = 0.189, *p* = 0.434] and a small effect size. The overlapping indices show a moderate overlap (0.677) and a small separation index (0.323).

#### Esteem

The COVID-19 group showed higher esteem values (*mean* = 17.912, *SD* = 5.485) than the non-COVID-19 group (mean = 13.71, *SD* = 6.441), and the difference was statistically significant [*t*_(64.366)_ = −2.899, *g* = 0.695, *p* = 0.005] with a moderate–big effect size. The overlapping and separation indexes were moderate at 0.546 and 0.454, respectively.

#### Coping

Considering resilient coping, there was no statistically significant difference [*t*_(64.732)_ = 0.085, *g* = 0.020, *p* = 0.932] between the COVID-19 (mean = 10.147, *SD* = 3.036) and non-COVID-19 groups (mean = 10.204, *SD* = 2.637). The effect size was negligible, the overlapping index was big (0.825), and the separation index was small (0.175).

#### Experience

The group of health professionals who worked in the COVID-19 ward reported a more positive experience (*mean* = 73.750, *SD* = 21.137) than the other group (*mean* = 52.647, *SD* = 22.537). The difference was statistically significant [*t*_(64)_ = −3.925, *g* = 0.954, *p* < 0.001), and the effect size was big. The overlapping and the separation indexes were moderate at 0.494 and 0.506, respectively.

Overall, in Study 2, higher psychological distress was associated with lower esteem (*r* = −0.33, *p* < 0.008) as well as with lower resilient coping (*r* = −0.34, *p* < 0.006). Moreover, higher esteem was associated with a more positive appreciation of work experience (*r* = 0.65, *p* < 0.001) and with resilient coping strategies (*r* = 0.50, *p* < 0.001).

## Discussions

This two-step research aimed to develop a new questionnaire to evaluate the psychological impact of COVID-19 on health professionals and compare the psychological experience of those who worked in the dedicated ward to those who continued working in ordinary departments.

According to Study 1, the EGA showed how the CPI-HP items constitute two well-distinct but correlated dimensions: psychological distress and esteem. Then, the EGA results were confirmed by the CFA reporting good fit indexes for the CPI-HP structural validity. The questionnaire showed good psychometric properties, representing a reliable and useful measure of psychological distress and esteem among health professionals. In particular, higher psychological distress was negatively associated with esteem, suggesting the protective role of esteem toward distress as in line with the TMT (Greenberg et al., 1986). Such a tool may be useful in clinical contexts to assess and monitor the health professionals' psychological health, encompassing both the negative and the protective factors.

Consequently, in Study 2, the CPI-HP was administered to assess and explore the psychological impact of COVID-19 on health professionals of a rehabilitation center during the pandemic of 2020. In particular, Study 2 results provided a reliable description and comparison of the psychological experience of health professionals who cared for post-acute patients with COVID-19 since the early phases of the pandemic, also comparing them with a group who did not directly work with patients with a COVID-19 diagnosis. Although all healthcare professionals reported a considerable level of psychological distress during the emergency, those who were not involved in the COVID-19 ward showed perceived lower esteem and lower appreciation of experience than those who worked in the COVID-19 ward.

Moreover, according to the overlap graphs and indexes, the two groups showed different distributions of scores in psychological measures, even if these differences were not evident by observing the means and Hedge's *g* values only (Pastore and Calcagnì, 2019). The measures with the greater differences between groups were found in esteem and positivity of experience—with the COVID-19 group reporting higher values. The distributions of psychological distress were not so different, but a larger part of the COVID-19 group reported lower distress when compared to the non-COVID-19 group whose distress values were more tending to higher scores—suggesting that all healthcare workers faced high distress during the pandemic regardless of the COVID-19 or non-COVID-19 ward. Finally, the two groups showed similar distributions in the resilient coping levels, suggesting its value as a resource for both groups.

Such psychological differences and similarities among the COVID-19 and non-COVID-19 groups should be considered to inform clinical support interventions.

Moreover, the psychological differences among groups may be explained in the light of the following considerations. First, those who worked in the COVID-19 ward perceived safer work conditions consisting of special suits and accessories and more severe hygienic practices, as reported by operators during routine équipe meetings with psychologists. Second, in the COVID-19 ward, a large structured team was constituted and met regularly to share decisions and practices. The members of the team also had a WhatsApp group to support each other. Third, the social acclamation made them feel a sense of heroism that probably contributed to coping better with the stressful aspects of their professional and personal lives. Briefly, being involved in a new, threatening, and challenging experience—so important at an (inter)national level—strengthened the organizational and individual resources.

On the other hand, those who did not work in the COVID-19 ward, although reporting coping resources not different from the other group, also suffered from strong psychological distress and reported a more negative work experience, together with less perceived esteem. These results may be due to the different organization of the non-COVID-19 ward where the individual protection devices (IPDs) were simpler and where people could not constitute a new group of work, regularly sharing decisions about practices and feelings. Moreover, they were not part of a highly socially celebrated work context, despite being recognized as heroes triggered both positive and ambivalent reactions (Hennekam et al., 2020).

Regarding the clinical meaning of these findings, it is likely that the work team with the higher group identification and esteem could also appreciate more a problematic experience despite the distress. Maybe distress would have been higher without these positive resources. According to the TMT (Pyszczynski et al., 2004), dangerous situations (i.e., COVID-19 emergency) generate distress that can be buffered through (self-)esteem that is rooted in one's role in society, work, and purpose in life. It is worth noting that all these aspects were salient for healthcare workers during the emergency. Moreover, recent literature showed that meaning in life can be found in work-related aspects, especially in traumatic and emergency situations as the COVID-19 pandemic (Nowicki et al., 2020). In a nutshell, feeling important and perceiving to have a meaningful role in society may have strengthened the esteem and the appreciation of the experience.

Some limitations can be acknowledged in the present work. Although sufficient to correctly estimate statistical parameters, future studies could increase the sample size to obtain even more robust results. Moreover, these studies were conducted in a single COVID-19 rehabilitation center; future studies could test the generalizability of these results to other circumstances (e.g., other infective diseases).

Although most of the literature highlighted the negative impact of COVID-19 for health professionals (Benfante et al., 2020; Pappa et al., 2020), this study is one of the few trying to also consider the positive and protective factors as esteem, resilient coping, and the positivity of the experience (Rieckert et al., 2021). Recent studies also showed that the COVID-19 pandemic implied a severe psychological burden for health workers, but also COVID-19 patients and caregivers and the general population as well (Bruno et al., 2020; Nese et al., 2020; Panzeri and Rossi Ferrario, 2020; Parola et al., 2020; Que et al., 2020; Rossi Ferrario et al., 2021). Despite this fact, a significant number of people avoided seeking social support and/or professional psychological help (Ratti et al., 2017; Rossi and Mannarini, 2019), probably due to the associated social and personal stigma (Mannarini et al., 2018, 2020; Mannarini and Rossi, 2019). Thus, large-scale psychological and social interventions should support individuals in these challenging circumstances.

Future research will deepen psychological reactions to stressful situations and evaluate the effectiveness of psychological interventions to promote functional psychological adaptation and resilience.

## Conclusions

Based on these findings, it is important to assess and monitor the psychological health of healthcare professionals in stressful circumstances as the COVID-19 pandemic, and the CPI-HP is proposed as a good tool to do so. Psychological screening programs should identify those operators who show a higher risk of (acute) stress reactions. Healthcare workers operating in either COVID-19 or non-COVID-19 wards similarly suffered from psychological distress, suggesting that timely psychological interventions should support them to reduce discomfort and symptoms. At the same time, resources to strengthen may include resilient coping processes and esteem.

## Data Availability Statement

The datasets presented in this article are not readily available because restrictions apply to the availability of this data to guarantee the respondents privacy. Requests to access the datasets should be directed to the corresponding author.

## Ethics Statement

The studies involving human participants were reviewed and approved by Scientific Reasearch Direction of the Scientific and Clinical Research Institute of Veruno, Maugeri Institutes. The participants provided their written informed consent to participate in this study.

## Author Contributions

AP did the analysis and interpretation of data. AP and SR participate in drafting the article. All authors made substantial contributions to conception and design, acquisition of data, revised the article critically for important intellectual content, and give final approval of the version to be submitted and any revised version.

## Conflict of Interest

The authors declare that the research was conducted in the absence of any commercial or financial relationships that could be construed as a potential conflict of interest.

## Figures and Tables

**Table A1:** Items of the questionnaire.

**Item #**	**Item text**
**Psychological distress scale**	
1	La mia alimentazione è stata più disordinate
	*My eating habits were more disordered*
2	Ho avuto sintomi come mal di testa, disturbi gastro-intestinali o altri disturbi fisici
	*I had symptoms such as headaches, gastrointestinal disorders, or other physical disorders*
3	Ho sentito più irritabilità/nervosismo
	*I felt more irritability and nervousness*
4	Ho avuto difficoltà a dormire
	*I had trouble sleeping*
5	Il mio umore è stato instabile
	*My mood was unstable*
6	La stanchezza che ho provato è stata particolarmente intens
	*The fatigue I felt was particularly intense*
7	Ho sentito più ansia del solito
	*I felt more anxiety than usual*
8	Ho avuto paura di non farcela
	*I was afraid I wouldn't make it*
**Esteem scale**	
1	Mi sono sentita/o valorizzata/o nel mio lavoro da parte dei miei cari
	*I felt appreciated for my job by my loved ones*
2	In questo periodo ho sentito una motivazione più forte al mio lavoro
	*In this period, I felt a stronger motivation in my work*
3	Ho sentito forte l'appartenenza a un gruppo di lavoro
	*I felt myself strongly belonging to a work team*
4	Mi sono sentita/o valorizzata/o nel mio lavoro da parte dei colleghi
	*I felt appreciated in my job by colleagues*
5	Ho dato più valore al mio lavoro
	*I appreciated my job more than usual*
6	Mi sono sentita/o valorizzata/o nel mio lavoro da parte dei superiori
	*I felt appreciated in my work by my superiors*
7	Ho sentito di partecipare a qualcosa di davvero importante
	*I felt like I was part of something really important*
